# Method for validating radiobiological samples using a linear accelerator

**DOI:** 10.1140/epjti2

**Published:** 2014-04-29

**Authors:** Muriel Brengues, David Liu, Ronald Korn, Frederic Zenhausern

**Affiliations:** Center for Applied NanoBioscience and Medicine, The University of Arizona College of Medicine, 425 N. 5th Street, Phoenix, AZ 85004 USA; Scottsdale Healthcare, Scottsdale Clinical Research Institute, 10510 N. 92nd Street, Scottsdale, AZ 85258 USA

**Keywords:** Linac accelerator, Blood samples, Biodosimetry, Gene expression

## Abstract

**Abstract:**

There is an immediate need for rapid triage of the population in case of a large scale exposure to ionizing radiation. Knowing the dose absorbed by the body will allow clinicians to administer medical treatment for the best chance of recovery for the victim. In addition, today’s radiotherapy treatment could benefit from additional information regarding the patient’s sensitivity to radiation before starting the treatment. As of today, there is no system in place to respond to this demand. This paper will describe specific procedures to mimic the effects of human exposure to ionizing radiation creating the tools for optimization of administered radiation dosimetry for radiotherapy and/or to estimate the doses of radiation received accidentally during a radiation event that could pose a danger to the public. In order to obtain irradiated biological samples to study ionizing radiation absorbed by the body, we performed *ex-vivo* irradiation of human blood samples using the linear accelerator (LINAC). The LINAC was implemented and calibrated for irradiating human whole blood samples. To test the calibration, a 2 Gy test run was successfully performed on a tube filled with water with an accuracy of 3% in dose distribution. To validate our technique the blood samples were *ex-vivo* irradiated and the results were analyzed using a gene expression assay to follow the effect of the ionizing irradiation by characterizing dose responsive biomarkers from radiobiological assays. The response of 5 genes was monitored resulting in expression increase with the dose of radiation received. The blood samples treated with the LINAC can provide effective irradiated blood samples suitable for molecular profiling to validate radiobiological measurements via the gene-expression based biodosimetry tools.

**PACS numbers:**

87.53.-j; 87.53.Bn

## Background

Since September 11, 2001, the possibilities of a radiological or nuclear terrorist attack have been a central focus for both governmental agencies and communities throughout the world. There is an urgent need to have the adequate infrastructure to rapidly assess radiation injury in such a mass casualty scenario. Biodosimetry measurements after a radiation incident will be an immensely helpful tool in order to perform screenings, triages and management of clinical facilities in a mass casualty incident [1]. In case of an accidental radiation exposure, the effects of ionizing radiation can be wide-ranging and involve either the entire body or specific organs. Depending of the dose received, different medical treatments can be adapted and in most cases, the deadly effects of high radiation exposure (>2 Gy) can be mitigated by early triage and treatment decision. Unfortunately, as of today, no single time point measurement that is diagnostic of radiation exposure can reliably or rapidly discriminate the different levels of radiation received. Different platforms are currently under development along with the discovery and evaluation of radiation induced biomarkers to achieve that same goal of being able to measure the dose of radiation absorbed by the body [2–5]. The dicentric chromosome assay (DCA) is currently considered as the goal standard method because of its high sensitivity and accuracy [6]. The dose estimation of this method is based on the frequency of radiation specific aberrant chromosomes with two centromeres (dicentrics) in an irradiated individual’s peripheral blood lymphocytes. But this assay is not applicable in mass casualty incident because it is very labor intensive and time consuming (several days) [7]. The γ-H2AX assay also used in radiation biodosimetry is a direct measure of the number of DNA double strand breaks (DSB) induced by ionizing radiation [8, 9]. The yield of γ-H2AX foci has been shown to be linearly related to dose over a very wide dose range. The number of foci per cell in macaque lymphocytes after total body irradiation with doses of 1, 3.5, 6.5 and 8,5 Gy increase linearly with the irradiation dose (especially at doses greater than 1 Gy) [8]. This assay gives a same day result, but requires that the blood samples are available within about 36 hours of irradiation. Gene expression based assay have been extensively used by many laboratories for biodosimetry measurement of radio-responsive genes in human peripheral blood lymphocytes and is a potential method that fits the criteria to provide high-throughput data with an accurate measurement and in a timely manner [10–18].

The monitoring of the dose administrated and received in radiotherapy is also needed as the possibility of radiation induced cancer exists for patients exposed intentionally to radiation. Being also able to measure the radio-sensitivity of each individual before starting radiotherapy treatment would be of great benefit not only to help control the late toxicity effect of treatment but also to integrate a personalized approach to tumor treatment based upon the chances of recovery and recidivism. One common tool needed for all of these studies is appropriate clinical samples in order to test the platforms under development. When studying the effects of ionizing radiation, it is not always possible to use *in vivo* samples. Several governmental research programs including NASA, Armed Forces, and BARDA agencies are pursuing work with non human primates. In addition to costs ranging in the millions of dollar and ethical issues, there is not really a standardized animal model in place to compare the results between studies [19]. While there is a need for animal models in radiation research, it might not always be necessary and not for every stage of the research. Very often cancer patients themselves are volunteering to participate in these studies to provide irradiated blood samples while on radiotherapy treatment.

The present study focused on the technique to provide irradiated samples to study biodosimetry. The main sources to irradiated samples are either a Gammacell-40 with a Cesium-137 source or a Cobalt-60 source, as well as X-rays or electron beams for a linear accelerator which does not contain radionuclide sources. The use of cesium-137 has been discontinued for practical reason and safety concerns in radiotherapy. Cobalt-60 is currently used in external beam radiotherapy devices found mostly in developing countries. Based on a report in 2006, there were several thousand radiotherapy devices in the United States in over 2,400 institutions and clinics [20]. Fewer than 250 cobalt-60 teletherapy devices are licensed in the United States and most of those are thought to be in storage for decay, in use for other purposes (such as fixed radiography), or in use for teaching. This is because the linear accelerator (LINAC) is considered a better, more accurate and versatile radiotherapy tool, and has largely supplanted cobalt-60 teletherapy devices in the United States and other developed countries [21]. Another advantage of the LINAC is that it does not require replacement of the radiation source such as the cobalt or cesium sources that have an issue with radioactive decay contributing to dose inhomogeneities and errors in dose calculation. The LINAC is the device most commonly used for external beam radiation treatments for patients with cancer. It is used to treat all parts/organs of the body by delivering high-energy X-rays or electron beams to the region of the patient’s tumor. These treatments can be designed in such a way that they destroy the cancer cells while sparing the surrounding normal tissue. More recently, the LINAC has been utilized to irradiate blood components [22]. Blood component irradiation is the only method of preventing a risk of transfusion associated graft versus host disease [23]. It has been demonstrated that by utilizing the LINAC, the internal irradiation procedures has been proven to be safe and feasible, and along with the significant cost/time reduction suggested that it is more advantageous than external procedures in hospitals without dedicated devices [22]. Our goal being to provide samples that will mimic real life situation as radiotherapy treatment, we irradiated our samples using the linear accelerator (LINAC). Using the LINAC for *ex-vivo* irradiation to study biodosimetry will allow a direct comparison with future data obtained from *in-vivo* irradiation. Comparing samples irradiated with the same source will eliminate false interpretations that could rise from using different irradiation methodologies.

This paper describes the process to perform irradiation on human blood samples using the LINAC in order to provide the appropriate irradiated samples as a tool for radiation biology study. To validate our method, the *ex-vivo* irradiated samples were analyzed to monitor the changes due to irradiation. We chose gene expression assay to analyze the data as it has been extensively used by many laboratories for biodosimetry measurement of radio-responsive genes in human peripheral blood lymphocytes, it has also been the method of choice in our laboratory for many years and it provides a result in less than 3 hours, however any assay could be used to analyze the data once the samples are irradiated with the LINAC. Validation of the use of LINAC can expand the opportunity to use this instrument for other biodosimetry studies and help to standardize the methodology for radiation trials in the future.

## Results and discussion

### LINAC calibration

**Figure 1:**
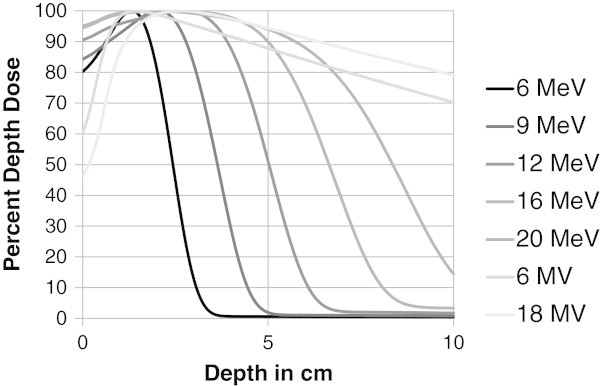
**Percent depth dose for all radiation beams from 21EX LINAC.** The field size is 20x20 cm for all beams. The percent depth dose for the lowest-energy beam is the left-most curve.

**Figure 2:**
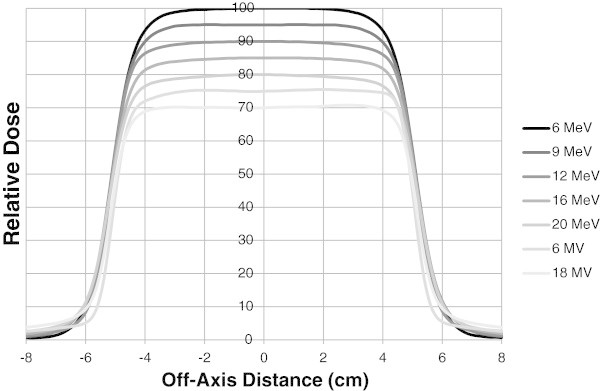
**In-plane dose profiles for all radiation beams from 21EX LINAC.** The profiles are for the depth of the maximum dose for each beam. From bottom to top curves are for 20 MeV, 16 MeV, 12 MeV, 9 MeV and 6 MeV profiles respectively.

Linear accelerators (LINACs) are the most popular equipment to deliver radiation therapy in hospitals. A Varian 21EX (S/N 1847) linear accelerator at the department of radiation oncology was used for the blood irradiation in this study. The Varian 21EX has two photon-beam energies (6 MV and 18 MV beams) and five electron-beam energies (6 MeV, 9 MeV, 12 MeV, 16 MeV and 20 MeV beams) that can be selected by the operator. A radiation beam was selected to provide the most homogeneous dose to blood samples. The depth dose curve shows the dose deposition along the beam direction and the radiation beam profile indicates the dose distribution perpendicular to the beam direction (Figures 1 and 2). These curves are for a field size of 20 cm × 20 cm and were measured in water. Our experimental setup was designed to have the radiation beam directed vertically down toward the floor at the blood tube holder that was lying horizontally on the table. With this geometry, we need to select a radiation beam with a uniform depth dose for about 1.5 cm (slightly more than the diameter of the tube) and a uniform profile for the central 5 cm (slightly longer than the length of the test tube). The radiation beam that best meets these requirements is 20 MeV electron beam at a depth of 2 to 3.5 cm (Table 1). The output of the radiation beam was calibrated based on the AAPM TG-51 protocol using a PTW Farmer-type ionization chamber (model 30013) (PTW, Freiburg, Germany) and a CNMC 1100 electrometer (CNMC Co, Nashville, TN) [24]. Both the ionization chamber and the electrometer were calibrated by Accredited Dosimetry Calibration Laboratory (ADCL) at the University of Wisconsin. Based on the calibration with this set up, the cone output factor for 20 cm cone with 20E is 0.953 cGy/monitor unit (MU) and the profile correction is 0.99. To deliver 2 Gy to the blood tube the required MU setting is 200/0.953/0.99 = 212 MU. To deliver 4 and 6 Gy, MU settings are 424 and 636 MU respectively. A rigorous quality assurance program has been already established to check the beam characteristics for this LINAC, including daily check with a Nuclear Associate QA phantom (Inovision Co. Carle Place, NY) and monthly calibration by a medical physicist certified by the American Board of Radiology using an ADCL-calibrated ionization chamber and electrometer. The outputs of all beams were also verified regularly by Radiological Physics Center at M D Anderson Cancer Center in Houston through mailed dosimeter program.

**Table 1:** **Dose variations due to depth doses and profiles for 21EX radiation beams**

	Depth of Max Dose (dmax)	Depth dose variation (dmax + 1.5 cm)	Profile variation (±4 cm from center axis )	Total variation
6 MV	1.4 cm	4.9%	2%	6.9%
18 MV	2.7 cm	3.3%	3%	6.6%
6 MeV	1.5 cm	86.5%	1%	87.5%
9 MeV	2.2 cm	49.3%	2%	51.3%
12 MeV	2.9 cm	19.7%	0.5%	20.2%
16 MeV	2.0 cm	2.5%	1%	3.5%
20 MeV	2.1 cm	0.4%	2%	2.4%

Total variation is the smallest for 20 MeV electron beam.

### Dose measurement on blank sample

**Figure 3:**
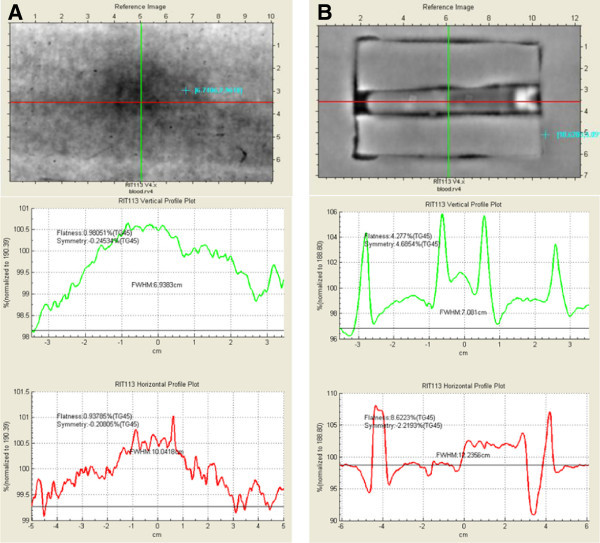
**Image of the scanning for EDR2 films from the top of the tube (A) and the side of the tube (B).**

The sample holder was made of Plastic Water® and Superflab® material, which may have slightly different radiation attenuation property from water. The introduction of the test tube, as well as the air space in and around the tube, further perturbs the dose distribution. Thermoluminescent dosimeters (TLD) and film dosimetry were used to study these effects. For this purpose, a blank measurement using a vacutainer tube filled with water and placed in the irradiation phantom was performed using LiF thermoluminescent dosimeters (TLDs) (Radiation Products Design, Inc. Albertville, MN) and EDR2 radiographic film (Carestream Health, Inc. Rochester, NY). TLD measures radiation dose by measuring the light output during the process of heating a crystal which has been exposed to ionization radiation. The amount of light emitted is dependent upon the radiation exposure. EDR films are often used to measure and verify the dose distributions. In addition, they are often used to perform various mechanical and dosimetric tests of the linear accelerator as part of routine quality assurance. Harshaw TLD-100 ribbons were taped around the tube (data not shown) and EDR-2 films were placed above and below the test tube (Figure 3). After irradiation with 212 MU of 20 MeV beam, TLDs were read with a Victoreen 2800 TLD reader and the EDR2 films were scanned with a Vidar 16 film scanner (Vidar Systems Corp., Herndon, VA). The radiation doses based on TLD results, which has an accuracy of 5%, were 2.03, 1.97 and 2.03 Gy for the top, bottom and the side of test tube respectively (data not shown). These readings were in agreement with the expected dose of 2 Gy. The dose profile along the vertical line is shown in the green curve; the one along the horizontal line is represented by the red curve. The dose distribution from the EDR2 film placed on the top of the test tube is quite uniform for both curves (Figure 3A). For the film placed below the test tube, there are very prominent dose inhomogeneities due to air gaps, tube cap and TLDs (Figure 3B). The shadow of the test tube is evident. The four peaks are due to air gaps surrounding the test tube and the filler Superflabs®. In addition to the air gaps at both ends, it also shows attenuation from the cap and the bottom of the test tube. Because the diameter of the cap is larger than the test tube, there is less water near the end of the cap, resulting in higher dose to the film at this end. Nonetheless, based on the film study, the dose pattern directly below the tube is within 3%.

### Gene expression analysis

**Figure 4:**
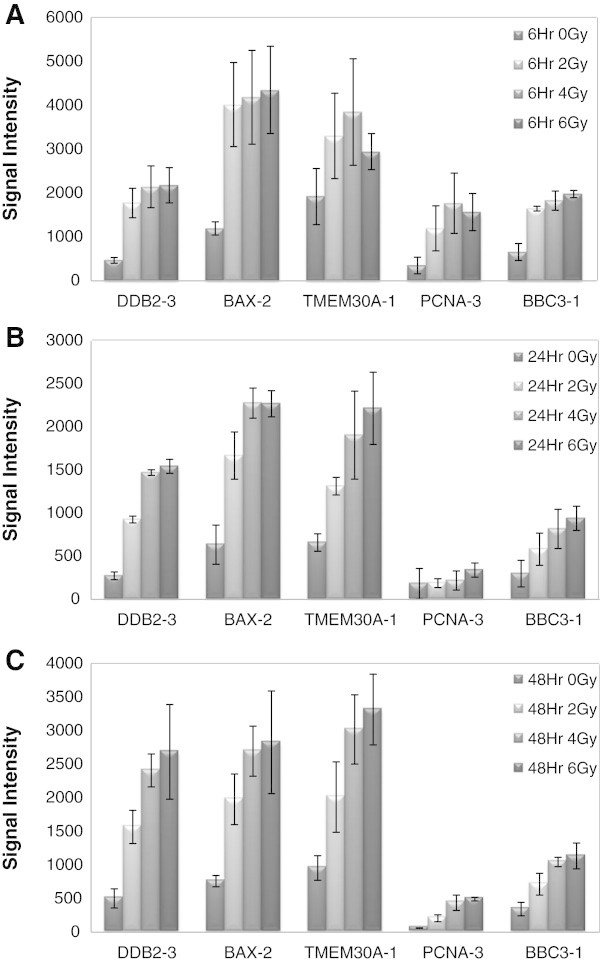
**CLPA gene expression profile from** ***ex-vivo*** **irradiated blood samples.** Whole blood samples were collected from 3 volunteers and irradiated at the doses indicated and analyzed with the CLPA assay. The transcript level of 5 genes (DDB2-3, BAX-2, TMEM30A-1, PCNA-3, BBC3-1) normalized to the housekeeping gene (GAPDH-4) was measured at 6, 24 and 48 hours post-exposure. The errors bars represent the standards deviation between the 3 volunteers.

The Chemical Ligation Dependent Probe Amplification assay (CLPA) is a Non Enzymatic Assay Technology (NEAT) based on a non-enzymatic oligonucleotide probe ligation method [25]. The assay enables for rapid, simple and inexpensive analysis of gene signature compatible with capillary electrophoresis (CE) system for rapid readout (e.g. multiple capillaries ABI 3130). Another advantage of the technology is its capability to work directly with whole blood samples which eliminate the need of RNA preparation and only require 25 μl of blood. Another reason for choosing this assay is the fact that the manufacturer specially formulated a commercial kit to measure gene expression for acute radiation exposure (http://redidx.com/index.php). The assay can be performed in less than 3 hours using equipment commonly found in research laboratories. The amount of each ligation product is proportional to the concentration of its RNA target sequence, and is proportionally amplified by PCR to allow quantification relative to the transcript of a housekeeping gene. The PCR products from the CLPA were analyzed on the CE instrument and the raw data from the CE were transferred into a statistical data spreadsheet file (e.g. Microsoft Excel) to provide the normalized data using the housekeeper (Figure 4). Blood samples from 3 volunteers were irradiated and we monitored the dose response after 6, 24 and 48 hours exposure of 5 genes DDB2-3, BAX-2, TMEM30A-1, PCNA-3 and BBC3-1 known as being biodosimetry markers [14, 15, 17]. These graphs represent the average signal intensity of the gene expression response of 3 volunteers with the error bars reflecting the standard deviation among the 3 individuals. It is important to mention that we are measuring the gene expression response from the leukocytes which vary in numbers from one person to the other (factor 3 among healthy individuals) which explain the size of the error bars. The trend of the expression pattern increased in response to radiation ranging from greater than 0 Gy to 6 Gy within the time periods of 6 hours, 24 hours and in particular 48 hours post-exposure where the gene signature signal remains stable and strong. For all the exposure times, a good dose response was observed between 0 and 2 Gy and also between 2 and 4 Gy for most of the genes. PCNA-3 is the lowest expressed gene measured with the CLPA assay among the 5 genes studied in this paper and when looking at the average signal for these 3 persons, a dose response was not observed as strong as if looking at the individual response (data not shown). If we look at the average signal without considering the standard deviation, we can see the dose response for PCNA-3 as well, as expected. For the dose response between 4 and 6 Gy, the differences among the signal intensity for all exposure times are much smaller than at lower doses or even nonexistent as it is for BAX-2. The fact that the separation by exposure dose is clearest between the lowest doses, with some overlap evident between the highest doses of 5 and 8 Gy has been reported previously in the literature [17]. In addition an overlap of the standard deviation is observed when the data is averaged over the 3 volunteers between 4 and 6 Gy, which indicates that such gene expression assay might not necessarily correlate with the cellular response at higher doses. The genes DDB2-3, BAX-2, TMEM30A-1, PCNA-3 and BBC-3 were anticipated to be up-regulated in response to radiation with the LINAC and it was observed that their expression increases in response to radiation between 0 and 2 Gy by a fold change up to 3.8 and between 2 and 4 Gy by a fold change up to 2.1. This data shows that the linear accelerator (LINAC) can be used as a characterization methodology providing *ex vivo* irradiation of blood samples to validate genomic-based radiation induced responses using assay chemistries suitable for small volumes processing.

## Conclusion

The authors described in detail how a LINAC at a standard Radiation Oncology Department from a hospital has been implemented in order to provide samples for validating the performance of a radiogenomic test that could be used for guiding medical countermeasures against acute exposure to ionizing radiations. A customized tube holder phantom was made to position the blood sample in a way that will mimic a body material heterogeneity and have an irradiation dose distributions and delivery process as accurate and similar as possible to an *in-vivo* irradiation. The LINAC was also calibrated in order to distribute the most homogenous dose to the sample tubes and the result showed a 3% variation in dose distribution. The phantom is actually designed to irradiate five tubes at a time but can be adapted to irradiate a larger number of tubes if needed.

By utilizing the LINAC installed in most radiotherapy departments, it is possible to provide irradiated samples that can be used to optimize and validate the radiosensitive biomarkers panel and assay chemistry platform for gene expression measurement. Not only does the use of the LINAC for that purpose allow for unlimited quantity of irradiated blood samples, it also avoids collecting extra blood from cancer patients and the use of animal models at multiple validation phases of the study. Our methodological approach using ex-vivo irradiation of blood samples could also be an advantage to evaluate relative biological effectiveness (RBE). The general procedure for evaluating RBE of most beam equipments is standardized with survival curves on cell culture. Using ex-vivo irradiation directly on blood samples instead of cell culture would allow accessing additional biomarkers representative of the biological interactions at the tissue or organ level. The use of the LINAC is a simple, fast and reliable way to provide *ex-vivo* irradiated samples with a similar dose accuracy as the one delivered during radiotherapy treatment on patients. In the future, we will assess samples from total body irradiation patients to validate various gene expression signatures and assay chemistries. Since the LINAC is the instrument used for radiotherapy treatment, these *in vivo* samples will have the same irradiation source as our preliminary study, which is important in order to compare data. The combined data will allow correlating the expression patterns of genes that are involved in biochemical and cellular responses to the irradiation dose by using the *ex-vivo* LINAC approach reported in this paper.

## Methods

### Design of experiment

Whole blood samples were collected from volunteers and *ex-vivo* irradiated at 0, 2, 4, and 6 Gy using the Varian Millennium Linear Accelerator (LINAC) with a customized phantom. The irradiated blood samples were placed in culture for allowing gene expression in response to radiation after 6, 24 and 48 hours. Following exposure, the samples were harvested and the samples were processed with the CLPA and the gene expression was analyzed on the CE instrument.

### Customized phantom for blood sample irradiation

**Figure 5:**
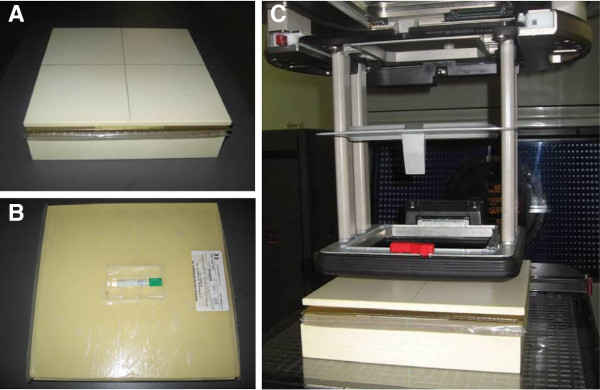
**Varian 21EX LINAC and customized phantom.** **A**. Phantom for irradiation of blood samples. **B**. Tube insert in 1.5 cm Superflab®. The two top layers of figure A were removed to show the test tube during the dry run. **C**. Set up of the phantom for blood irradiation with the Varian 21EX LINAC.

To facilitate and standardize the irradiation of multiple blood tube vacutainers using a linear accelerator, a custom phantom was build, made of Plastic Water® (CIRS, Norfolk, VA) and Superflab® slabs (Mick Radio-nuclear, Mount Vernon, NY) (Figure 5A and B). The Superflab® material is flexible and would not crush the blood tube. A slab of 5 cm of Plastic Water® was used as base; for the top, a 1.5 cm Superflab® slab with a hole cut at the center was created to accommodate the blood tubes (Figure 5B). Another 1 cm of Superflab® was placed to cover the blood tubes. Finally, another 1 cm of Plastic Water® was put on top of the 1 cm Superflab®. To irradiate the blood tubes vacutainers, the phantom is placed on the treatment couch with its center aligned with the central axis of the radiation beam at 100 cm SSD (Figure 5C).

### Blood collection, irradiation and culture

These experiments were approved by the institutional review board (IRB#2008-036) and were conducted according to the principles expressed in the Declaration of Helsinki. Written consent for participation in the study was obtained from all the subjects voluntarily. A total of 3 healthy donors, 1 male and 2 female between the age of 21 and 55 years old participated in this study. On the day of blood irradiation, informed consent was obtained from the volunteers. Prior to phlebotomy, a single certified radiation physicist programmed the Varian 21 EX (3100 Hansen Way, Palo Alto, CA 94306) at the following settings.: 20 MeV, 20 cone, and the following Monitor Unit (MU): 212, 414 and 636 MU for 2, 4 and 6 Gy. The blood irradiation phantom was put in the center of the table and raised to 100 cm source to surface distance (SSD). Samples containing 4 ml of peripheral blood samples were collected from healthy volunteers in glass vacutainer tubes (12.35 mg Sodium Citrate, 2.21 mg Citric Acid) (VWR International, Pittsburg, PA). The vacutainers were transported by batches of 5 for irradiation. The blood was exposed to 0, 2, 4 and 6 Gy X-rays using the Varian 21EX LINAC. After irradiation, blood samples were diluted 1:1 with RPMI 1640 medium (Invitrogen) supplemented with 10% heat inactivated fetal bovine serum (Invitrogen) and incubated for 6, 24 and 48 hours at 37°C in a humidified incubator with 5% CO2. After the indicated exposure times, the sample tubes were removed from the incubator and set up in a sterile field. The blood samples were aliquoted in 2 ml Eppendorf tubes and an equal volume of CLPA reaction buffer was added (1:1). The samples were stored at 4°C.

### Gene expression assay – chemical ligation dependent probe amplification (CLPA)

**Figure 6:**
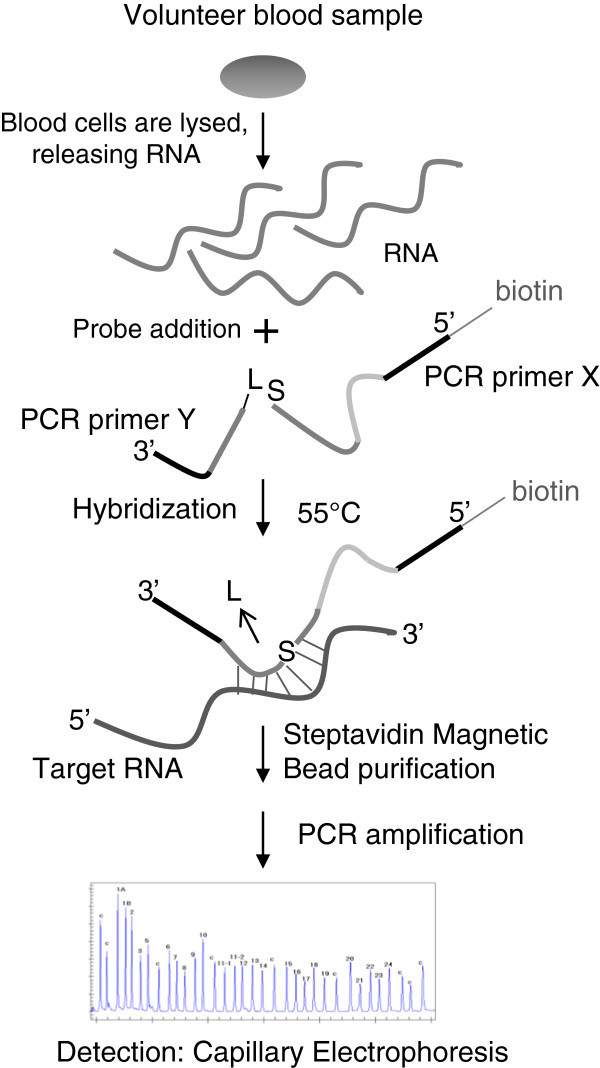
**Description of the CLPA protocol.** CLPA uses chemical ligation chemistry to generate target specific reaction products which contain a 5′-biotin group to allow for easy capture and purification of the ligated product, universal primer sites to allow for multiplex amplification by PCR that is specific for only the ligated product, and identifier sequences that allow for easy readout using existing instrumentation. Two probes (S and L) are designed for each target RNA species. The S-probe contains a 5′ biotin moiety, a universal upstream primer sequence (black), a target hybridizing domain (red), and a target identifier domain (blue) to enable separation for subsequent measurement. The L-probe contains a 5′-leaving group (L), a hybridizing domain (red), and a sequence complementary to a universal downstream primer (black). Upon hybridization to adjacent sequences in the target RNA, the nucleophilic group on the S-probe reacts with the L-probe to displace the leaving group and ligate the two oligonucleotides. The ligation products from many probe pairs are produced in proportion to the concentrations of their corresponding targets, and are then isolated by capture on streptavidin-coated magnetic particles, and amplified by PCR. The PCR products are then analyzed on a capillary electrophoresis system.

We selected a commercially available assay chemistry test (CLPA, DxTerity Diagnostics, Rancho Vista, CA) for multiplex gene expression analysis that combines a robust chemical ligation process and a sample stabilization method (Figure 6) (http://dxterity.com/dx_direct.php). The assay requires 25 μl of whole blood which corresponds to 100 μl of the blood sample obtained previously. For each reaction, 20 μl of S probe (probe with a nucleophillic group) mix was added to the 100 μl blood sample and placed in a thermal cycler for 10 min at 80°C. During the incubation, a master mix containing 60 μl CLPA Buffer 2 and 20 μl L-probe (probe with an electrophillic leaving group) mix per sample was prepared and kept at 55°C. The master mix was immediately added to each sample and gently mixed before incubation for 3 hours at 55°C for hybridization. When the hybridization was done, 4 μl of M-270 Streptavidin Dynabeads was added to each sample and incubated for 5 min at 55°C. Three washes were then performed using a magnetic block for 10 sec and 100 μl CLPA wash buffer. A complete PCR mixed was prepared (20 μl per reaction) by combining an equal volume of PCR primer mix (Dxterity) and 2X Dynamo probe master mix (New England Biolabs, F-450 L). After the final wash, the beads were re-suspended in 20 μl CLPA complete PCR solution and the PCR was performed using the following cycling conditions: 94°C for 10 min followed by 30 cycles [94°C for 10 sec, 60°C for 20 sec, 72°C for 20 sec]. 1 μl of PCR product was used to perform the analysis on the multiple capillaries electrophoresis (CE) system (ABI 3130 instrument, Life Technologies, CA).

## Authors’ information

MB: Research Associate Scientist – Biologist – University of Arizona.

DL: Medical Physicist responsible for patient radiotherapy treatment delivery at Scottsdale Healthcare.

RK: Radio oncologist and Chief Medical Officer and Director of Virginia Piper Cancer Center at Scottsdale Healthcare.

FZ: Professor of Basic Medical Sciences - University of Arizona College of Medicine and Director of the Center for Applied Nanobioscience and Medicine (ANBM) at the University of Arizona.
